# Prolonged Treatment with Inhaled Corticosteroids does not Normalize High Activity of Matrix Metalloproteinase-9 in Exhaled Breath Condensates of Children with Asthma

**DOI:** 10.1007/s00005-015-0328-z

**Published:** 2015-02-04

**Authors:** Katarzyna Grzela, Wioletta Zagorska, Alicja Krejner, Malgorzata Litwiniuk, Anna Zawadzka-Krajewska, Aleksandra Banaszkiewicz, Marek Kulus, Tomasz Grzela

**Affiliations:** 1 Department of Paediatrics, Pneumonology and Allergology, Medical University of Warsaw, Warsaw, Poland; 2 Department of Histology and Embryology, Medical University of Warsaw, Chalubinskiego 5, 02-004 Warsaw, Poland; 3 Potgraduate School of Molecular Medicine, Warsaw, Poland; 4 Department of Paediatric Gastroenterology and Nutrition, Medical University of Warsaw, Warsaw, Poland

**Keywords:** Asthma, Exhaled breath condensate, Inhaled steroids, MMP-9, Remodeling

## Abstract

The airway remodeling in asthma is associated with increased amount of matrix metalloproteinase (MMP)-9. High levels of MMP-9 were found in mucosal biopsies, sputum and in exhaled breath condensates (EBC) of asthma patients. However, there are no data concerning real in vivo activity. Inhaled corticosteroids are effective in asthma control, but it is unclear, whether they only attenuate inflammation, or also protect against progressive remodeling of respiratory tract. Therefore, the aim of the study was to assess the amount and activity of MMP-9 in context of pro-inflammatory cytokines (IL-6, IL-8 and tumor necrosis factor, TNF), measured in EBC of asthma-suffering children, treated with inhaled steroids. The study involved 27 children with asthma, continuously treated with inhaled fluticasone propionate, and 22 healthy controls. In addition to routine clinical screening, the selected cytokines in EBC were analyzed using Ultrasensitive ELISA, whereas activity of MMP-9 was assessed using a novel immunozymography method. Despite chronic treatment with inhaled steroids mean MMP-9/EBC activity in asthma group was significantly higher than in healthy controls. Moreover, high MMP-9/EBC in asthma-suffering children significantly correlated with IgE serum levels. The IL-6 and IL-8 concentration was below the detection limit in all EBC samples. TNF/EBC levels were similar in both, asthma and healthy children. We hypothesize that MMP-9 hyperactivity in asthma may be closely related to high IgE serum levels. Our results suggest that inhaled steroids may be ineffective to prevent asthma-associated airway remodeling. Finally, we emphasize the necessity of further research focused on MMP-9 inhibition in asthma treatment.

## Introduction

Matrix metalloproteinases (MMPs) belong to the family of zinc-dependent proteases, which are necessary for physiological turnover of extracellular matrix and tissue repair (Crosby and Waters 2010; Hadler-Olsen et al. 2011). However, when overexpressed, MMPs, especially MMP-9, were found to be engaged in development of cancer metastasis, delayed wound healing and some vascular diseases (Birkedal-Hansen et al. 1993; Hadler-Olsen et al. 2011; Klein and Bischoff 2011). Recently, the involvement of MMP-9 has also been postulated in pathomechanism of respiratory tract diseases, which are associated with progressive remodeling of the airway wall (Atkinson and Senior 2003; Salib and Howarth 2003). The mentioned diseases include chronic obstructive pulmonary disease and allergic asthma (Holgate 2009; Shapiro 2009). Since patients with advanced asthma reveal increased concentration of MMP-9 in blood, sputum and bronchoalveolar lavage (BAL), it is assumed that MMP-9 may play the pivotal role in that remodeling (Cataldo et al. 2002; Lee et al. 2001; Lemjabbar et al. 1999). Moreover, it has been found that MMP-9 levels correlated with severity of asthma symptoms (Karakoc et al. 2012; Mattos et al. 2002). Thus, it was postulated that this association may be useful in clinical practice to monitor asthma exacerbation and/or effectiveness of treatment (Cataldo et al. 2002). Nevertheless, despite reported differences in MMP-9 levels, the clinical usefulness of MMP-9 assessment in BAL and sputum seems to be limited. The first limitation concerns the fact, that in patients with severe asthma or asthma exacerbation, the collection of BAL may be difficult or even impossible. The second obstacle is due to relatively high baseline concentrations of MMP-9 in the saliva (Raitio et al. 2005). According to that, much larger differences in absolute values of enzyme levels between analyzed sputum samples are required to be of statistical significance. Therefore, an interesting solution could be a novel noninvasive diagnostic approach based on the collection and biochemical assessment of exhaled breath condensates (EBC) (Gagliardo et al. 2009; Gessner and Wirtz 2010). Most recently, there have been reported increased MMP-9 levels in EBC of patients with asthma (Barbaro et al. 2014; Karakoc et al. 2012). However, since analyzing the MMP-9 protein level, instead of measurement of its activity, mentioned reports did not provide data sufficient for the assessment of real clinical relevance of this finding. For that reason, an actual in vivo activity of MMP-9 in EBC of asthma-suffering individuals still needs to be determined.

There is still a debate, whether inhaled corticosteroids, which are widely used to control the asthma symptoms, are really effective in protection against progressive remodeling of the respiratory tract (Bisgaard et al. 2006; Mattos et al. 2002; Todorova et al. 2009). Therefore, the aim of the present study was to assess the amount and the actual activity of MMP-9 in EBC of asthma-suffering children, which were continuously treated with inhaled steroids. To estimate the inflammatory status of the respiratory system, selected pro-inflammatory cytokines, interleukin (IL)-6, IL-8 and tumor necrosis factor (TNF), were also measured in condensates of exhaled air. These data were then compared with respective results obtained in healthy control group.

## Materials and Methods

### Patients

Our study involved 27 children (11 female and 16 male, mean age 12.6 ± 3.4) with chronic allergic asthma. The asthma recognition was based on the recommendations of “GINA”—the Global Initiative for Asthma, Strategy for Asthma Diagnosis and Prevention (updated 2009, available from http://www.ginasthma.org). The children from study group have been receiving inhaled glycocorticosteroids with a daily dose 500 µg of fluticasone propionate for at least 6 months. They were allowed to take second-generation H1 antihistamines and inhaled β2-mimetics (upon request).

The control group comprised 22 healthy individuals, 10 female and 12 male (mean age 12.4 ± 4.9), attending the outpatient clinic of the Department of Paediatrics, Pneumonology and Allergology, at the Warsaw Medical University, for routine healthcare control visits.

All individuals and their parents signed the informed written consent to participate in the study. The protocol of this experiment was formally approved by the local bioethics committee (Approval No. KB/246/2012).

According to the inclusion and exclusion criteria, all children were subjected to a routine clinical examination and laboratory tests. The peripheral blood morphology was analyzed using flow cytometry, with determination of total leukocyte count and main leukocyte subpopulations. To confirm the allergic background of asthma, the total IgE serum level and specific IgE or skin tests were performed. The asthma control was verified by the presence of clinical symptoms, spirometric assessment and exhaled nitric oxide (eNO) level (Smith et al. 2005; Zeiger et al. 2006). The NO concentration in exhaled air (eNO) was measured using Sievers NO 280 device (GE Analytical Instruments, Boulder, CO, USA). The results were expressed as ppb (particles per billion) units.

The spirometry was performed using Lung test 1000 device (MES, Krakow, Poland). The results of assessment of forced expiratory volume in the first second (FEV1) and forced vital capacity (FVC) were calculated as the FEV1/FVC ratio (Tiffeneau–Pinelli index). After adjustment to the patients age, the data were shown as the standard deviation score. The reference values were based on data from large multicenter population studies (Hankinson et al. 1999; Quanjer et al. 2010; Zapletal and Chalupova 2003).

### EBC Assessment

The EBC collection was performed using ECoScreen condenser (Jäger, Höchberg, Germany), according to protocol described previously (Zagorska et al. 2013, 2014). In brief, after 15–20 min of adaptation to ambient condition, the 15-min-long EBC collection was performed. Samples of breath condensate (approx. 700–1,000 μl each) were immediately deep frozen and stored at −70 °C, until being used for further analysis.

#### Cytokines

The cytokine concentrations in EBC were estimated in duplicates, using Human IL-6, IL-8 and TNF ultrasensitive ELISA kits, respectively, according to detailed protocols provided by the manufacturer (all tests from Invitrogen, Camarillo, CA, USA). The absorbance of analyzed samples was measured using the Microplate Reader 550 (BIO-RAD, Hercules, CA, USA). The OD results were converted to the specific cytokine concentrations (expressed in pg per ml of EBC), based on the respective standard calibration curves. For all tested cytokines the assay sensitivity, corresponding to the lowest points of the standard calibration curves, was 0.1 pg/ml.

#### MMP-9

The MMP-9 activity and concentration were estimated in EBC samples using QuickZyme Human MMP-9 activity assay. This novel immunozymography method allowed an assessment of both, the specific protein level, as well as its actual enzymatic activity. The test was performed according to detailed protocol provided by the manufacturer (QuickZyme BioSciences, Leiden, Netherlands). All EBC samples were run in two series, each of them in duplicates. The first series reflected the actual amount of active MMP-9 in exhaled air. The second group of samples was pretreated with p-aminophenyl mercuric acetate (APMA) to ensure the assessment of the entire MMP-9 concentration (including both, the active MMP-9 and silent pro-MMP-9 form) in tested sample. The baseline absorbance of analyzed samples was measured with the Microplate Reader 550 (BIO-RAD) immediately after addition of substrate (T_0_) and then after 2 h of incubation at 37 °C (T_2_). The MMP-9 concentrations in EBC, both, active and total, were calculated based on the standard calibration curve of APMA-activated human recombinant MMP-9 and expressed in ng/ml. The assay sensitivity (0.01 ng/ml) corresponded to the lowest point of the standard calibration curve.

The possible contamination of collected breath condensates with the saliva was verified by the assessment of the amylase concentration in ten randomly selected EBC samples from each group (Gaber et al. 2006; Zagorska et al. 2014).

### Statistical Evaluation

All the parameters tested in the study were compared between both groups using Mann–Whitney *U* test. The relationship between analyzed parameters was estimated by two-tailed Spearman correlation test. For both assessments, the differences were considered as statistically significant at *p* < 0.05.

## Results

Clinical characteristics of both groups, results of blood tests, eNO concentration and spirometric assessment are summarized in Table 1.

**Table 1:** The clinical characteristic of patient groups

Parameter/patient group	Asthma (*n* = 27)	Healthy control (*n* = 22)
Age (years)	12.6 ± 3.4	12.4 ± 4.9
Sex distribution (female/male)	11/16	10/12
Polysensitized patients	22^a^	0
Monosensitized patient	5^a^	0
Including patients sensitized to:		
Grass	23	0
Mites	22	0
Animals	15	0
*Alternaria*	5	0
*Cladosporium*	3	0
FEV1_SDS_	0.5 ± 0.2	0.3 ± 0.2
FEV1/FVC_SDS_	−0.3 ± 0.2	−1.1 ± 0.5
Exhaled NO (ppb)	27.2 ± 14.3^a^	12.8 ± 5.3
Blood eosinophils (×10^3^/μl)	0.31 ± 0.1	0.18 ± 0.1
Total IgE serum level (kU/l)	1,302 ± 758^a^	33.2 ± 9.8

Mean values ± SD

*FEV1* forced expiratory volume in the first second, *FVC* forced vital capacity, *FEV1/FVC* Tiffeneau–Pinelli index, *SDS* standard deviation score (Z-score), − value corrected in relation to the age

^a^Statistically significant, as compared to control group

**Fig. 1:**
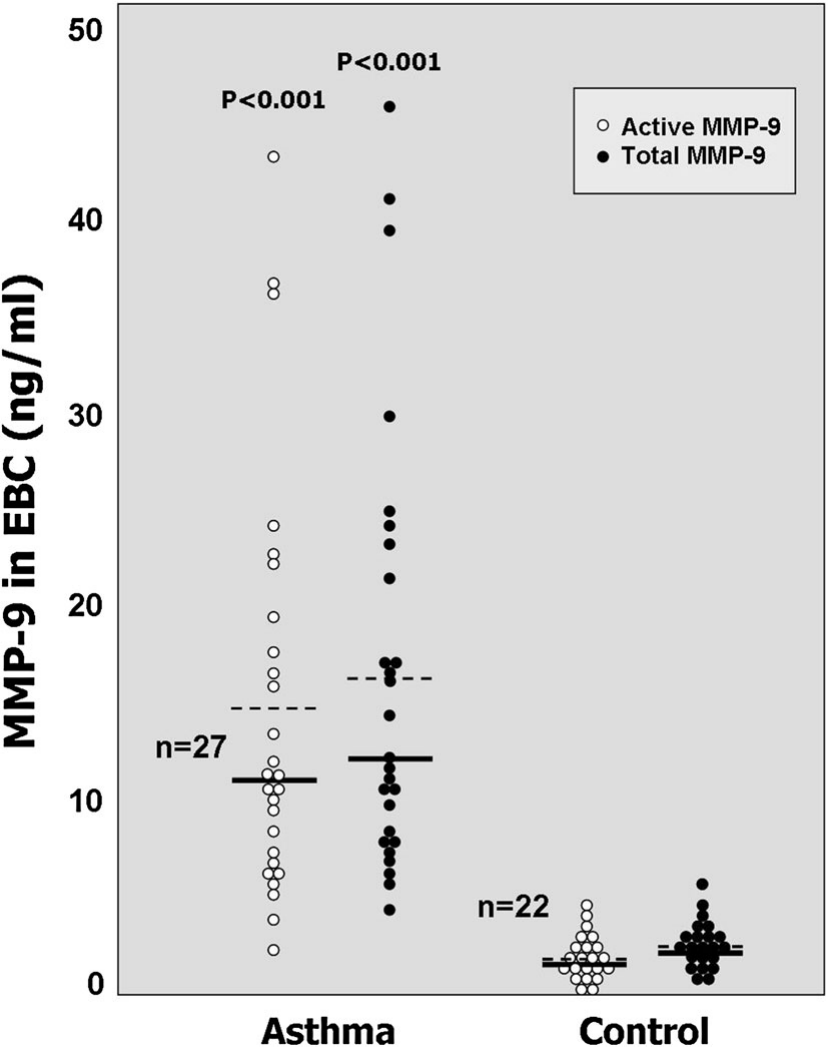
The concentration of MMP-9 in exhaled breath condensate (EBC) samples was expressed in ng/ml as an active (*white dots*) or total enzyme level (*black dots*). *Each dot* represents the result of respective measurement in one individual from asthma or control groups. Mean values of MMP-9 concentration in each group were indicated as *dashed lines*, the median values were shown as *solid lines*. The *p* values below 0.05 were considered as statistically significant (by Mann–Whitney *U* test)

The mean concentrations of MMP-9 in breath condensates of patients in asthma group were statistically significantly higher than in healthy controls. The mean concentration of active enzyme was 14.7 ± 10.5 ng/ml in asthma group vs. 2.0 ± 1.1 ng/ml in healthy controls. Similarly, the mean concentration of total MMP-9 (composed of both, inactive pro-enzyme and its active form) was 16.1 ± 10.9 ng/ml in asthmatic patients vs. 2.1 ± 1.1 ng/ml, in control individuals (Fig. 1). The ratio of active enzyme to the total amount of MMP-9 protein was slightly higher in asthma group than in healthy controls (91.9 vs. 81.3 %, respectively), however, this difference did not reach statistical significance.

**Fig. 2:**
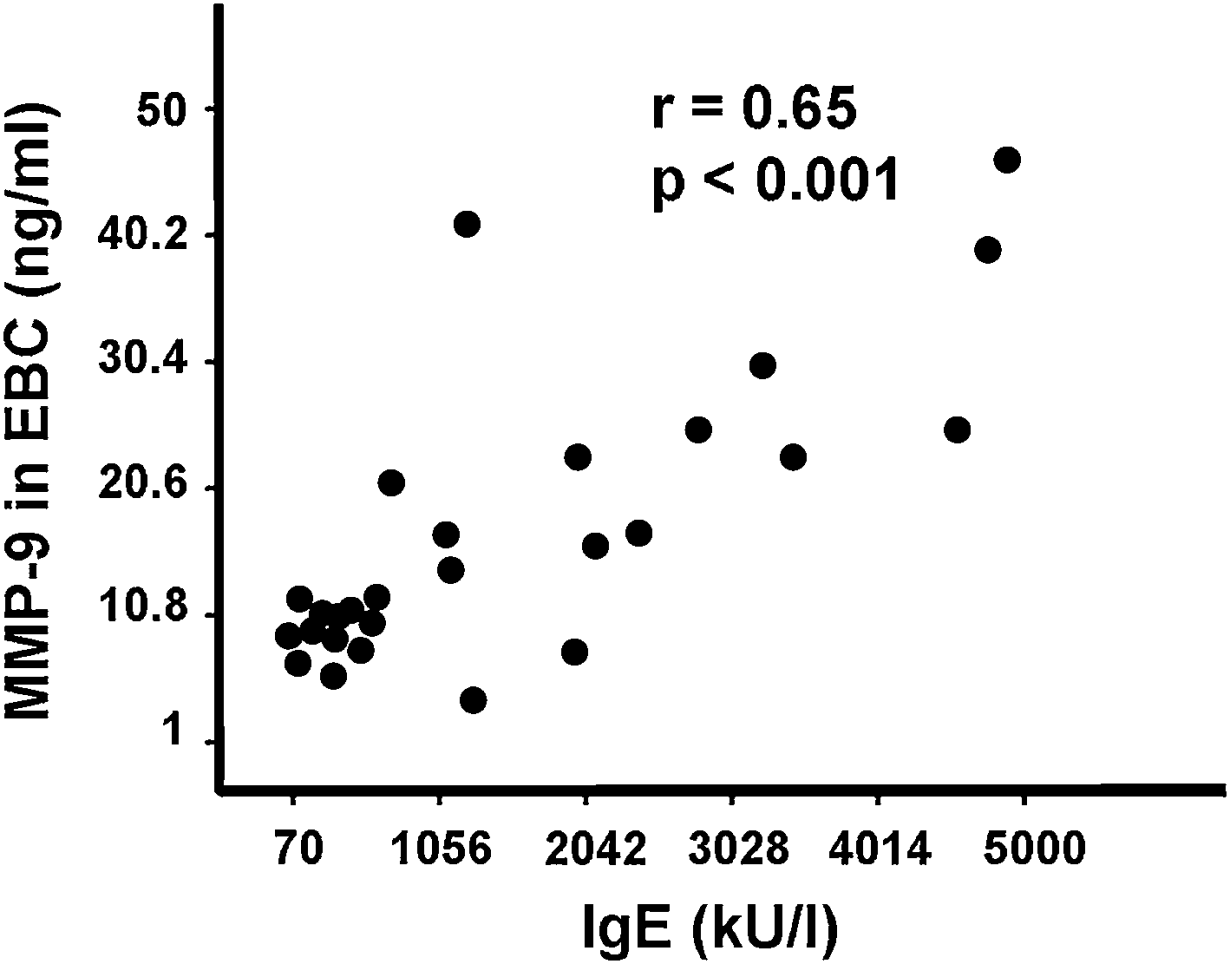
The relationship between concentration of active MMP-9 in EBC (expressed in ng/ml) and serum levels of total IgE (in kU/l). *Each dot* corresponds to the result of respective measurement in one individual from asthma group. The *p* value below 0.05 was considered as statistically significant (by Spearman correlation test)

Interestingly, it has been found that the concentrations of both, active and total MMP-9 in EBC samples revealed statistically significant correlation (*r* = 0.65 and *r* = 0.63, respectively, at *p* < 0.001) with the total IgE serum levels (Fig. 2). Based on mild clinical symptoms in asthma-suffering patients, the allergic bronchopulmonary aspergillosis has been excluded as possible reason of high IgE levels in that group.

**Fig. 3:**
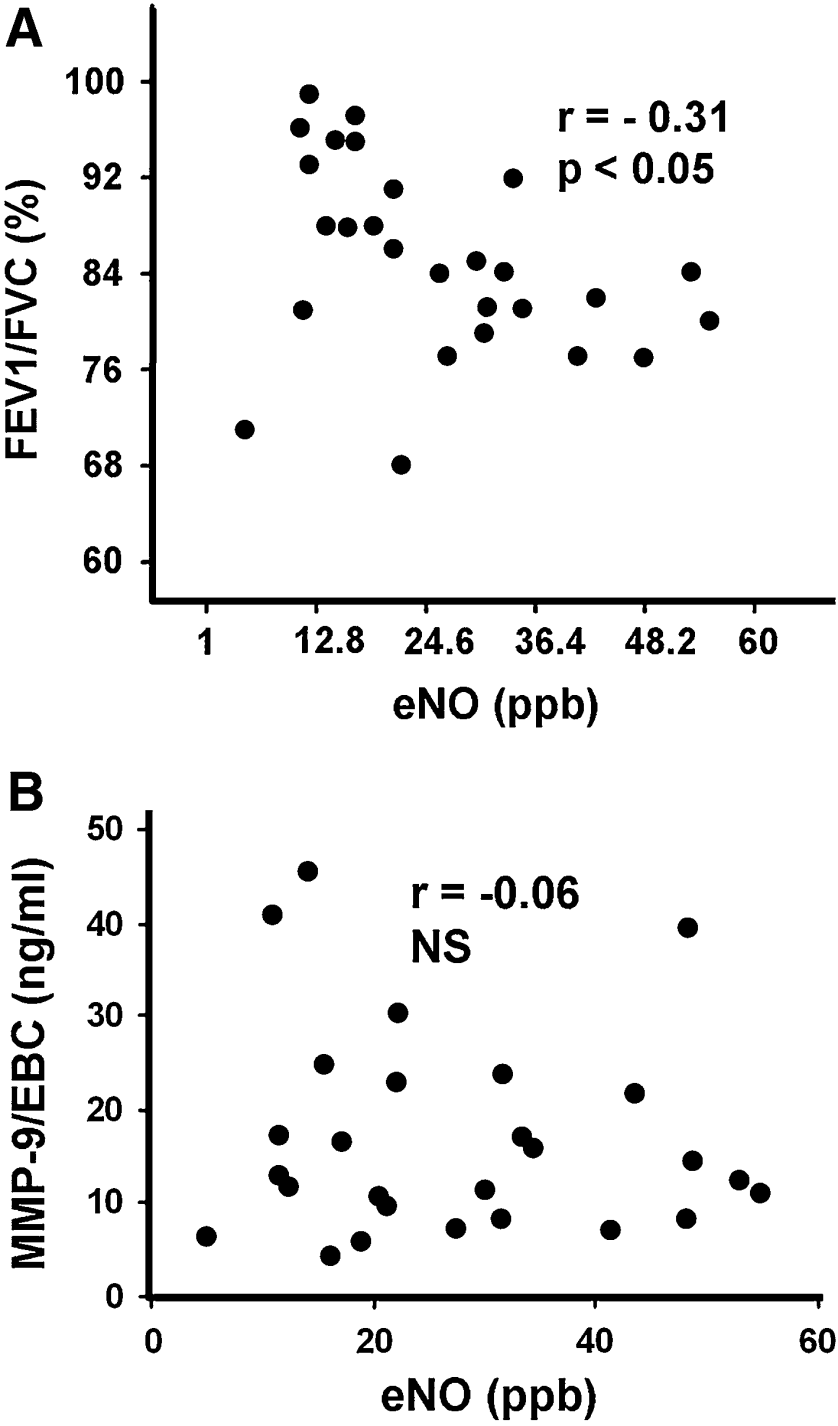
The relationship between exhaled nitric oxide (expressed in particles per billion, *ppb*) and Tiffeneau–Pinelli index (expressed as FEV1/FVC %) (*upper panel* **a**) or active MMP-9, assessed in exhaled breath condensate (*EBC*) and expressed in ng/ml (*lower panel* **b**). The *p* value below 0.05 was considered as statistically significant (by Spearman correlation test). *NS* non-significant

There was observed a weak negative correlation between Tiffeneau–Pinelli index (expressed as FEV1/FVC %) and levels of exhaled nitric oxide (*r* = −0.31, *p* < 0.05; Fig. 3a). However, none of both mentioned variables revealed any significant correlation with MMP-9 levels (Fig. 3b).

The concentrations of IL-6 and IL-8 in all EBC samples of both, patients and control groups, were below the detection limit, i.e., <0.1 pg/ml. In contrast to them, TNF was detectable in all tested samples. The mean concentrations of TNF/EBC were 3.45 ± 0.87 pg/ml in asthma group and 3.74 ± 0.71 pg/ml in control individuals, but they did not differ statistically.

## Discussion

Our study has shown for the first time that children with stable asthma, when compared to healthy controls, had significantly elevated active form of MMP-9 in their EBC. This observation may have practical value, as a noninvasive measurement of MMP-9/EBC activity is easy to perform even in very young children. Therefore, it may potentially be useful to assess current state of disease. Interestingly, the activity of MMP-9/EBC in our patients was higher than in health controls, despite relatively mild symptoms of asthma, possibly due to prolonged treatment with inhaled corticosteroids. It has been proven that the allergic asthma may be well controlled pharmacologically in majority of patients (Kroegel 2009). In fact, also in our patients the inhaled corticosteroids allowed satisfactory control of asthma clinical symptoms, with exhaled NO levels only slightly higher than normal limit. Moreover, the concentrations of IL-6, IL-8 and TNF in EBC of steroid-treated patients were very low and similar to those of healthy controls. These finding could support the assumption that in our patients the inflammatory reaction was at least significantly reduced.

It is well known that neutrophils and macrophages are main source of MMP-9 in inflammatory reaction (Delcaux et al. 1996; Mautino et al. 1997). However, their significant contribution to the origin of high MMP-9 in our steroid-treated asthmatic patients, although cannot be excluded, remains unconvincing. This statement was based on low concentrations of mentioned inflammatory markers, including exhaled NO levels. Therefore, in our patients, some alternative sources of MMP-9 should also be considered, e.g., associated with IgE-dependent pathway. This assumption was developed from the observation that increased MMP-9/EBC activity in our asthma group significantly correlated with high levels of total IgE. Actually, this last finding may suggest at least three potential alternative sources of MMP-9. Obviously, the first candidate may be Fcε-RI-expressing mucosal mast cells, which are known to produce and release various proteases, including MMP-9, upon IgE-mediated and allergen-independent stimulation (Kimata et al. 2006; Maxová et al. 2010). The second source of this enzyme could be mast cells interacting with neighbor fibroblasts. In this cooperation, mast cells utilize their receptors for IgE as sensors, whereas fibroblasts work as effector component of the system to produce and release the MMP-9 (Abel and Vliagoftis 2008). Finally, the third possibility may be smooth muscle cells in respiratory tract, which have been shown to express both, Fcε-RI and Fcε-RII types of IgE receptors on their surface. It has been proven that IgE-mediated stimulation of airway smooth muscle cells resulted in activation of the same pathways (Roth et al. 2013), which are involved in regulation of the MMP-9 expression in those cells (Liang et al. 2007). It is plausible that in proposed IgE-dependent mechanism, each of mentioned cell types may serve as an important source of MMP-9. Furthermore, the observed correlation between total IgE and MMP-9/EBC may also have another practical implication. It could explain the rationale to implement anti-IgE treatment in prevention strategy against asthma-associated remodeling (Buhl 2005).

The mentioned above results may suggest that inhaled steroids, although effective to satisfactorily control clinical symptoms of asthma, do not normalize the initial high activity of MMP-9 in respiratory tract. Thus, inhaled steroids seem to have limited influence on natural course of asthma in children and, as shown by other authors, do not protect against airway remodeling (Bisgaard et al. 2006; Guilbert et al. 2006). Therefore, high MMP-9/EBC activity associated with an increased total IgE level could presumably be considered as a risk marker of airway remodeling and poor clinical prognosis in asthma course, but this issue still requires further studies.

It is noteworthy that some authors have reported decrease of MMP-9 concentration following the inhaled steroid treatment (Wang et al. 2011; Weitoft et al. 2014). However, conclusions from mentioned studies were based on measurement of MMP-9 protein amount, without considering its actual enzymatic activity. The use of novel MMP-9-specific immunozymography technique enables to assess both—total protein level and its activity (Grzela et al. 2011). Obviously, this method provides more reliable data, which reflect in vivo status, including actual function of MMP-9 molecule inside the respiratory tract. Accordingly, since it is difficult to extrapolate those results to our system, we intend to verify them using described above methodology in ongoing prospective trial.

Although MMP-9 is recognized as key factor involved in asthma-associated structural changes of airway, detailed mode of its action remains unclear. The well-known mechanism concerns proteolytic degradation of extracellular matrix components and basement membrane, which facilitate the active leukocyte passage and their accumulation in respiratory tract wall. However, MMP-9 may also modulate inflammatory reaction by interference with cytokine/chemokine network, e.g., via catalytic activation of IL-8, release of latent transforming growth factor-β1 and, possibly, IL-13 (Atkinson and Senior 2003; Mehra et al. 2010; Van den Steen et al. 2000). Therefore, although the exact role of MMP-9 in asthma still needs to be elucidated, the measurement of its activity in EBC may provide new data supporting the value of MMP-9 as indirect progression marker in the assessment of airway remodeling.

Finally, in addition to the usefulness of MMP-9/EBC measurement as a risk marker, the confirmation of pivotal role of MMP-9 in asthma-associated remodeling may have another potential benefit. It strongly supports the need for further research focused on implementation of modulators of MMP-9 activity also in asthma treatment. That approach was already demonstrated to be clinically effective in vascular diseases associated with MMP-9 hyperactivity—aortic aneurysm and chronic wounds healing (Curci et al. 1998; Grzela et al. 2008, 2014; Nagashima et al. 2002). Recently, neovastat (AE-941), natural MMP-9 antagonist, has been shown to display some beneficial properties in murine model of asthma (Lee et al. 2005). Therefore, possibly the MMP-9 inhibition may be considered as a method of pharmacological prevention against respiratory tract remodeling in asthma. However, as discussed in details elsewhere, this perspective still requires extensive research.
